# Interaktionen mit soziotechnischen Systemen lernen: Geragogische Perspektive auf die Bedeutsamkeit von Wechselwirkungen und Beziehungen in Lernprozessen mit Älteren

**DOI:** 10.1007/s00391-023-02274-0

**Published:** 2024-01-25

**Authors:** Renate Schramek, Stefanie Engler

**Affiliations:** 1 Hochschule für Gesundheit Bochum Gesundheitscampus 6, 44801 Bochum, Deutschland; 2 Ev. Hochschule Freiburg, Freiburg, Deutschland

**Keywords:** Geragogik, Menschen, Technology, Psychosoziale Unterstützung, Relationales Bildungsverständnis, Geragogy, Humans, Technology, Psychosocial support, Relational understanding of education

## Abstract

**Hintergrund:**

Der Ansatz soziotechnischer Systeme (STS) für ein gelingendes Leben im Alter richtet den Fokus auf die Verbindung technischer und soziokultureller Faktoren in einem. Technologische Systeme sind längst nicht mehr auf einfache Funktionen beschränkt; vielmehr ermöglichen sie individualisierbare Einsätze sowie interaktive Nutzung, verbunden mit komplexen Kommunikations- und Handlungssträngen. Die verbundenen Interaktionen mit dem technischen System legen die Perspektive auf Wechselwirkungen und die Beziehung nahe.

**Ziel des Beitrags:**

Forschungsergebnisse zeigen, dass individualisierbare Funktionen sozialtechnischer Systeme, bezogen auf Akzeptanz und Nutzung, von Älteren positiv bewertet werden. Aus geragogischer Perspektive stellen sich Fragen zur Aneignung der Älteren und zur Gestaltung der Lernprozesse (soziotechnischer Lernarrangements).

**Material und Methoden:**

Entwickelt wird eine relationale Sicht auf Lern- und Bildungsprozesse rund um Aneignung und Handlungskompetenzerwerb zur Gestaltung mit und zur Nutzung von STS in realweltlichen Situationen, basierend auf einem formativ angelegten qualitativen Forschungsdesign des Projekts „RUBYDemenz“. Speziell werden Daten/Ergebnisse aus Interviews mit freiwillig tätigen Lernbegleitenden, professionell tätigen Praxisbegleitenden, Fokusgruppen und Forschungswerkstätten herangezogen.

**Ergebnisse und Diskussion:**

Von der Empirie lassen sich Ansatzpunkte für Bildungs- und Lernprozesse zur Kompetenzerweiterung im Kontext von Techniknutzung entwickeln. Ansätze zu Aus‑, Fort- und Weiterbildung schließen an, ebenso wie die Frage, ob der Forschungs‑/Entwicklungsprozess selbst als Lernprozess angelegt sein muss, in den alle Beteiligten als Lernende involviert sind, mit dem Ziel, eine bestmögliche Passung der technischen Lösungen mit den avisierten Einsatzbereichen in realweltlichen Situationen zu erreichen.

## Abstract (en)

**Background:**

The approach of socio-technical systems (STS) for a successful life in old age focuses on the combination of technical and sociocultural factors in one. Technological systems have long since ceased to be limited to simple functions; instead, they enable customizable applications, interactive use combined with complex communication and action strands. The associated interactions with the technical system suggest a perspective on interactions and relationships.

**Objective:**

Research results show that individualizable functions of STSs are rated positively in terms of acceptance and use by older people. From a geragogical perspective, questions arise regarding the appropriation of older people and the design of learning processes (socio-technical learning arrangements).

**Material and methods:**

A relational view of learning and educational processes relating to the appropriation and acquisition of competences for designing and using STS in real-world situations is developed based on a formative qualitative research design of the “RUBYDemenz” project. In particular, data/results from interviews with voluntary learning facilitators, professional practice facilitators, focus groups and research workshops are utilized.

**Results and discussion:**

Empirical findings can be used to develop starting points for educational and learning processes to expand competences in the context of technology use. Approaches to education, training and further training follow on from this as does the question of whether the research/development process itself must be designed as a learning process in which all participants are involved as learners, with the aim of achieving the best possible fit between the technical solutions and the intended areas of application in real-world situations.

Der Ansatz „soziotechnischer Systeme“ (STS) verbindet einen sozialen Faktor (Person, Gruppe) und einen technischen Faktor (z. B. Robotik) zu einem neuen Gesamtsystem. Es ergibt sich eine Beziehung mit Wechselwirkung zwischen den Teilsystemen. Wechselwirkung und Beziehung rücken in den Fokus [10, 14]. Aus gerontologischer und geragogischer Sicht werden Fragen betrachtet zu

1. Integration gängiger Technologien in soziale Systeme (wie z. B. den Haushalt Älterer oder in die Häuslichkeit von Menschen mit Demenz und ihren Angehörigen etc.),
2. Wechselwirkung und Interaktionen zwischen Mensch und Technologie,
3. Qualität von Lernbegleitung, als Beitrag zum Gelingen der Aneignungsprozesse.

Die Verbindung technischer und soziokultureller Aspekte in der Technikentwicklung bietet viele Gestaltungsoptionen für ein gelingendes Leben im Alter. Technische Systeme (TS) werden in der Alltagsgestaltung, zu Unterstützung, Kommunikation, Information, Entlastung und Strukturierung eingesetzt [21]. Mensch und TS treten in eine Folge von Interaktionen, die auf einem komplexen Zusammenspiel von Handlungsplanung der Beteiligten basieren. Zusätzlich wirkt teils auch in die Systeme integrierte künstliche Intelligenz ein, beispielsweise in dem STS RUBYDemenz (Infobox; [12]). Empirische Daten aus diesem Forschungsprojekt werden zur Verdeutlichung der folgenden geragogischen Überlegungen herangezogen.

## Infobox RUBYDemenz

Im STS RUBY, entwickelt im Projekt „RUBYDemenz“ (BMBF 2019–2023), interagiert eine Roboter-Puppe mit individualisierbaren Funktionen und personalisierten Informationen zu den Nutzenden mit Menschen mit Demenz und pflegenden Angehörigen in realweltlichen Situationen (Häuslichkeit). Nutzungsintentionen sind Unterstützung, Unterhaltung, Anregung, Strukturierung im Alltag [25, 27].

Neben dem TS kommt eine psychosoziale Begleitung der Nutzenden durch je 2 eigens auf diese Aufgabe der Einführung und Nutzung vorbereitete „RobotBegleitende“ (RB) zum Einsatz. Sie haben in einer Schulung gelernt, wie sie Techniklernen, ausgehend von eigenen Anliegen und bezogen auf die Nutzung durch Menschen mit Demenz, initiieren können, und wie sie die Menschen mit Demenz und die Angehörigen begleiten. Ihrerseits werden die freiwilligen RB durch Professionelle bei der Aufgabe begleitet. Die „RB“ führt das TS bei den Nutzenden durch einfache Erklärungen, Ermöglichen von Erfolgserlebnissen, gemeinsame Übungen mit der Puppe und Ausprobieren ein, regt auch durch eine positive Atmosphäre Techniklernen an und sorgt durch Übung und Wiederholung für Sicherheit im Tun. Neben technischen Fragen werden alle weiteren Anliegen der Nutzenden besprochen.

Das qualitativ angelegte Forschungsdesign zu Techniklernen und zum Einsatz der Robotik in den Familien umfasste die Ausbildung von 27 freiwillig tätigen RB im Alter von 26 bis 75 Jahren; diese brachten die Puppe im Alltag von 8 Familien zum Einsatz. Die Forschung zur Wechselseitigkeit im Lernprozess bezieht sich auf 4 Fokusgruppen mit RB, 7 halbstrukturierte Interviews mit RB, 2 halbstrukturierte Interviews mit Professionellen sowie 4 Forschungswerkstätten (FW) mit freiwilligen und professionellen Begleitenden zu Fragen nach förderlichen Lernbedingungen. Die Wechselwirkungsprozesse zwischen Mensch und ST wurden im Hinblick auf förderliche und hinderliche Bedingungen untersucht.

Individualisierbare und personalisierbare Funktionen eines Roboters [8, 19] ermöglichen einen individuellen TS-Einsatz [11]: Ausgangspunkt sind die Bedarfe und Motive Nutzender [23]. Biografische Daten, individuelle Vorlieben etc. können mit dem TS verknüpft werden; Kommunikation und Interaktion erfolgen mit persönlichem Bezug; Handlungsstränge bei Nutzung sind mit einer auf die Beziehung zwischen Mensch und TS gerichteten Intention verknüpft [20]. Die Nutzung von STS im Alltag und Zuhause erfordert zahlreiche Lernprozesse, bezogen auf die Entwicklung, Bedienung, Nutzung und die Handlungsgestaltung im Alltag [17]. Bei Nutzenden und ihren Angehörigen werfen die STS viele Fragen auf, z. B. Datenspeicherung, Datenschutz oder Datenverarbeitung und daran anschließende ethische Aspekte betreffend. Unter anderem wurde die Frage gestellt: Kann mich jemand durch die Kamera der Puppe sehen?

## Geragogische Positionen zu Alter(n), Bildung und Digitalisierung

Zur Annäherung an Digitalisierungsprozesse in Alter(n)skontexten hat ein Autor*innenkollektiv des Arbeitskreises (AK) Geragogik geragogische Positionen formuliert [1]. Demnach seien zu beachten:

1. die *Heterogenität* des Alters und *Teilhabeoptionen* aller (Bedeutung individualisierbarer Funktionen),
2. die stetige, kritische *Reflexion* von Chancen, *Risiken* und ethischen Implikationen (Begleitung und Moderation von Lernprozessen),
3. ein auf *Handlung* und aktiv Lernende gerichtetes Bildungsverständnis, um fehlende Technikkompetenzen/digitale Kompetenzen zu überwinden und die Fähigkeit zum selbstbestimmten Leben stärken zu können,
4. die Befähigung zur *bewussten Entscheidung *von (Nicht‑)Nutzung, die Notwendigkeit einer tiefen Auseinandersetzung mit TS/STS,
5. *Belastungs- und Zumutungsgrenzen* bei der Nutzung. Techniknutzung darf menschlichen Kontakt nicht ersetzen, die Funktionalität TS solle dem Menschen dienen,
6. es braucht *Lern- und Begleitungskonzepte*, die die genannten Aspekte umsetzen, auch im Kontext zivilgesellschaftlicher Potenziale,
7. inter-/transdisziplinäre *(Forschungs)Ansätze*, die Ältere als Co-Produzenten einbeziehen, um TS/STS entsprechend ihren Anliegen zu entwickeln,
8. verlässliche *Strukturen* zur Befähigung Älterer.

Für die Entwicklung, Evaluation, Einführung und Nutzung von STS gelten die skizzierten Aspekte besonders. Sie werfen Fragen auf, wie Techniklernen gelingen kann [9], wie Ansätze und Bedingungen dafür zu gestalten sind [26], welche Technikkompetenzen für STS erforderlich sind, und wie STS in Lehr-Lern-Settings eingebettet werden können [22]. In der Konsequenz folgen daraus die Notwendigkeit der Förderung digitaler Kompetenzen im Alter (I), ein auf die Besonderheiten von STS gerichtetes Bildungsverständnis (II) und die konsequente Umsetzung (III).

## Geragogische Annahme I: digitale Kompetenzen

Dem Ansatz der Medienkompetenz von Baacke folgend [2], implizieren Technikkompetenzen eine kognitive Dimension (Wissen zu Technik), eine Handlungsdimension (Nutzung), eine Gestaltungsdimension (mit Technik gestalten) und eine kritische wertbezogene Dimension (ethische Fragen; [4]). Geragogische Ansätze zielen auf:

- *Wissen* über TS/STS, einschließlich des Wissens, wofür diese, bezogen auf den eigenen Lebenskontext und im eigenen Alltag, genutzt werden können,
- *Fähigkeiten*, um TS/STS entsprechend den Funktionen aktiv und passiv im eigenen Lebenskontext, im Alltag gemäß eigenen Anliegen zu nutzen,
- *Gestaltendes Handeln* mit TS/STS selbst und gemeinsam mit anderen,
- *Techniksouveränität* als Urteilsfähigkeit über TS/STS sowie die Folgen, Fähigkeiten einer kritischen Auseinandersetzung, Reflexion und Positionierung zu persönlichen und gesellschaftlichen Folgen.

Mit Technikkompetenz haben Ältere die Option, eigene Lebensbereiche mithilfe von TS handelnd zu gestalten und TS/STS für eigene Anliegen, zu eigener Entlastung, Unterstützung einzusetzen und bei Einschränkungen für mehr Autonomie zu nutzen [15, 16]. Ein an den Anliegen der Älteren ausgerichtetes Vorgehen ist angezeigt, auch um (gemeinsam mit anderen) Handlungen mit TS/STS zu vollziehen sowie individuelle Fragen, Wiederholungen und begleitete Lernfortschritte zu ermöglichen [24].

## Geragogische Annahme II: ein relationales Lern- und Bildungsverständnis

Der geragogische Fokus geht über eine nur auf das Individuum zentrierte Sicht hinaus: Lernen/Bildung werden als Option verstanden, allein, in Gemeinschaften und sozialen Kontexten zu lernen sowie soziale Beziehungen und Verhältnisse mitzugestalten [22], im Sinne aufeinander bezogener, dialogischer Vorgänge. Die Bezogenheit in Lernprozessen, als Aspekt von Wechselseitigkeit, wird konzeptualisiert [23]: Lernen ist auch beziehungsorientiertes, „relationales Geschehen“; ein Prozess, der sich durch das „Ineinander von Selbst‑, Welt- und Anderenrelation ereignet“ [18]:

1. Beziehungsorientierung ist ein wesentliches Merkmal von Lernprozessen.
2. Erlebte Beziehungsqualität ist ein zentraler Faktor für Offenheit, die Zuwendung zu Neuem, zur kritischen Reflexion eigenen Verhaltens und zur Veränderung.
3. Im Sinne von „Eingebundenheit“ ist sie bedeutsam für die Lernmotivation.

Der Ansatz einer relationalen, auf Wechselwirkung angelegten Bildung gibt Anregungen für ein vertieftes Verständnis von „Begleitung“ als „Lernbegleitung“ [3].

## Geragogische Annahme III: Begleitung als Basiskonzept

Begleitungsansätze sind ein zentrales Format der Geragogik: In vielen geragogischen Feldern wurden sie erprobt, evaluiert und weiterentwickelt [3]. Eigens qualifizierte Freiwillige können individuelle und kollektive Lernprozesse anstoßen. Nach dem relationalen Verständnis umfasst Begleitung Räume, in denen sich Reflexion, Lernen und Begegnung vollziehen. Der Prozess ist „unverfügbar“, „nicht planbar“ und macht eine neue Qualität des Lernens und Problemlösens erlebbar [6, 7]. Beim Lernen mit und zu STS wird auch Technik/Robotik Teil und Akteur im Lernprozess [23]. Die interaktive Technologie/interagierende Robotik ist Akteur im Rahmen der Begleitungsräume. Dem „Handeln“ des TS kommt eine bisher so nicht beachtete Bedeutung zu. Bereits bei der Entwicklung der Funktionen müssen Wechselwirkungen und Reaktionen auf das TS mitgedacht werden; unbedacht können in der Mensch-Technik-Interaktion und im Lernen ungewünschte Folgen aufscheinen.

Diese geragogischen Grundannahmen werden im Folgenden beispielhaft mit den empirischen Ergebnissen zu Einführung und Nutzung des STS RUBY hinterlegt, verdeutlicht und zusammengeführt.

## Technikkompetenz für soziotechnische Systeme als Zukunftskompetenz – am Beispiel RUBYDemenz

In der heutigen Zeit ist es möglich, Kulturtechniken, Literalität durch Technik zu kompensieren, z. B. über Sprachfunktionen oder spezifische Software. Dies erfordert aber Technikkompetenzen. Daher sind diese selbst zur Kulturtechnik avanciert. Und auch ältere Menschen benötigen heute mehr Technikkompetenz zur Gestaltung des eigenen Alltags. Oft ist die Lebensgestaltung auf TS bezogen, und zugleich bieten TS Optionen zu Lebensgestaltung, Teilhabe, Unterstützung, Entlastung in allen Lebenslagen. Die Nutzung setzt aber „funktionierende“ TS voraus. Andernfalls sind Frustration und entsprechende Nichtnutzung wegen mangelnder Funktionalität empirisch belegt [26]. Technikkompetenz erfordert Wissen zu Technik und Bedienbarkeit, Handlungskompetenz, bezogen auf die Gestaltung sowie eine kritische Reflexion der Folgen und des Umgangs mit Technik. Diese Lernoutcomes erfolgen (im Alter) nicht nebenbei, sie müssen gezielt gelernt werden [26]. Im Kontext von STS kommen die Kommunikation und die Interaktion mit dem TS hinzu, woraus weitere Anforderungen erwachsen. Freiwillige Lernbegleitende beschreiben: „Du weißt sonst gar nicht, was kann die Puppe [Roboter], wie reagiert sie, wie sprichst du die an, wenn du das üben kannst, ist das sinnvoll“ (TRB_2 Z 279ff). Kompetenzen, Wissen wurden aufgebaut und in sicherem Lernraum wiederholt geübt (Funktionieren der Sensoren, der Roboter-Puppe, Kommunikation und Interaktion mit derselben, App-basierte Kommunikation zwischen Roboter-Puppe mit einem mobilen Endgerät etc.). Da die Roboter-Puppe im Alltag als „Vertrauter“ [22] wahrgenommen wird, sind zudem eine kritische Positionierung und Reflexion ethischer Aspekte erforderlich.

## Soziotechnische Systeme und Relationalität: Phänomen der Wechselseitigkeit in Interaktion von STS und Person

Die Lernprozesse zu STS erfordern eine grundlegende Betrachtung von Wechselseitigkeit und Beziehung. Wechselseitig meint eine gegenseitige Einflussnahme aller Akteure (das TS ist ein Akteur) im Lernprozess. Verhalten und Reaktion sind aufeinander bezogen.

Am STS RUBY wird deutlich, wie Interaktion mit einem Roboter stattfindet: „die fragt mich nach meinem Namen, das glaubt mir keiner“, so eine Nutzende überrascht. Freude, Enttäuschung, weitere Reaktionen auf das Verhalten eines TS im STS sind als Interaktion zwischen Mensch und anderen Beteiligten als Variable in den Blick zu nehmen. Die Gestaltung des Lernens ist davon geprägt, diese Wechselseitigkeit so einzubinden, dass sie sich nicht als Hemmnis erweist. Strünck et al. beschreiben die „co-constitution“ von Altern und Technologie in häuslichen Pflegesettings: „[…] the puppet can make persons with dementia and their family caregivers change communication about their relations. On the other hand, it is trained human companions that make vulnerable persons and their caregivers try out and thereby change the very functioning of technology“ [27].

Aufgrund der Komplexität und Wechselseitigkeit von STS ist die Gestaltung von Lernprozessen in der Mensch-Robotik-Interaktion eine anspruchsvolle Herausforderung des Technologielernens. Entsprechend muss es bereits in der Entwicklung und später in der Nutzung darum gehen, Aspekte der Wechselseitigkeit zu erkennen und emotional gefärbte (positiv und negativ erlebte) Sequenzen und Wirkungen in der konkreten Handlung vorauszusehen und in der Nutzung auszugleichen, z. B. bei einer enttäuschten Feststellung wie „Wieso sagt die jetzt nichts? Die kann ja gar nichts.“, so der Kommentar eines Nutzenden. Auch sind neben technischen Aspekten die Prinzipien der Wechselseitigkeit und die Beziehung zu Robotik, Lernpartnern etc. zu beachten. Lernbegleitende können, so im Projekt RUBYDemenz, vertraute Partner werden, und der Roboter-Puppe wurde überwiegend die Bedeutung eines Freundes oder Familienmitglieds zugeschrieben.

Die Reaktionen auf TS variieren: Von Neugierde, Interesse, Offenheit über Langeweile und Enttäuschung sind vielfältige Reaktionen zu nennen, so z. B. bezogen auf das RUBYSystem. Ebenso reagieren Menschen verschieden auf die von TS gesetzten Impulse. So muss die spätere Gruppe Nutzender bei der Entwicklung, Gestaltung, Anwendung angemessen einbezogen sein. Bei zielgerichteten Entwicklungen für spezifische Gruppen, z. B. mit wesensverändernden Erkrankungen, müssen die verbundenen Besonderheiten bedacht werden. Dies umfasst Absicht der Interaktion, Wechselwirkungen und Beziehung von Mensch und TS. Sie sollten interdisziplinär/interprofessionell betrachtet und diskutiert werden.

## Der Beziehungsaspekt in STS: Beziehung von Mensch und TS

Im Alter erfolgen Lern- und Bildungsprozesse oft als Reaktion auf „Irritation“ oder werden durch ein bestimmtes Motiv ausgelöst. Im Projektkontext RUBYDemenz zeigte sich dies z. B. daran, dass ein Zustand im Alltag zu Hause mit der Demenz nicht länger als zufriedenstellend oder hinnehmbar erlebt und mehr Strukturierung im Ablauf des Alltags gewünscht wurde oder Neugierde, bezogen auf die Innovation Roboter-Puppe, als Motivation benannt wurde. Hinsichtlich der Feststellung, Lernen ereignet sich in Beziehungen, gilt daher: Beim Einsatz von STS sind sowohl die Beziehung zu Lernpartner*in, Lernbegleiter*in, Lerngruppe zentral, und eben auch zu dem TS; besonders, wenn Letzteres auf Kommunikation und Interaktion ausgerichtet ist. Denn in die Lernprozesse fließen Erleben, Erfahrungen ein, beeinflusst oder ausgelöst durch Wechselwirkungsprozesse. Sie wirken sich auf das Lernen und das Lernergebnis aus.

Techniklernen in Form von Aneignungsprozessen (Wissen, Bedienbarkeit, Nutzung, Erlangen einer kritischen Sichtweise) und Kompetenzentwicklung (Erkennen der Gestaltungsoptionen und Handlungskompetenz) umfasst hier besonders ein Neu‑, Um- und Verlernen bisher gewohnter Handlungspraktiken –, und es ist auch bei einer bestehenden Demenz möglich. Da der Beweis dafür, dass auch bei einer vorliegenden Demenzerkrankung Lernen möglich ist, relativ neu ist, soll neben den Ergebnissen des RUBYDemenz-Projektes auch auf die Dissertation des schwedischen Wissenschaftlers Elias Ingebrand verwiesen werden, der ebenfalls anhand von Techniklernen aufzeigt, dass eine Demenzerkrankung nicht das Ende des Lernens bedeutet [13].

In RUBYDemenz sollte das durch die Demenz veränderte häusliche Leben durch den Einsatz des TS verändert werden, um mehr Struktur und Abwechslung im Alltag zu ermöglichen. Bezogen auf die Erkenntnis, dass Lernen in Beziehungen stattfindet, gilt: Das Lernen des STS RUBY ist auf Kommunikation und Interaktion ausgerichtet; diese Aspekte werden beim Lernen unmittelbar angesteuert. Erleben und Erfahrungen stehen im Mittelpunkt, werden in Wechselwirkungen im Lernprozess ausgelöst und beeinflusst. Das STS beeinflusst das Lernen und das Lernergebnis. Techniklernen, Aneignungsprozesse und Kompetenzentwicklung umfassen ein Neu‑, Um- und Verlernen gewohnter Handlungspraktiken.

Die Forschungsergebnisse zeigen: (Re)Aktionen des TS lösen Reaktionen in Verhalten, Denken und Kommunikation aus. Umfasst ist ein Spektrum von Freude, Sympathie, Enttäuschung, Irritation, Langeweile, Ärger etc. (ausführlich; [23]). Die emotionalen Zustände und die Art des Erlebens wirken sich auf das Denken, die Motivation zur Nutzung und das Lerngeschehen aus. Weitere Irritationen können in der Person, im Alltag der Nutzenden oder im TS liegen [23]. Ältere Menschen erleben evtl. Unsicherheiten gegenüber technischen Innovationen; sie können sich hemmend auf den Lernprozess auswirken [9]; auch das ist ein Grund, warum Lernbegleitung von Bedeutung ist.

## Soziotechnische Systeme und Lernbegleitung

Interaktionen von Älteren oder Demenzerkrankten in einem STS erfordern eine Lernbegleitung, die das Erleben und die Erfahrungen einordnet und das Lernen moderiert (Ergebnis FW2). So können Missverständnisse korrigiert und Schwierigkeiten bei der Nutzung überwunden werden. Dies ermöglicht, wenig konstruktiv wirkende Aspekte in den Lernprozess zu integrieren oder diese auszugleichen, z. B. wenn enttäuscht festgestellt wird: „Wieso sagt die jetzt nichts? Die kann ja nichts.“, damit diese sich weniger oder nicht hemmend auswirken [23]. Grundsätzlich gelingt das Techniklernen, wenn der Prozess durch die gemeinsame Formulierung *individueller Lernpfade* gestaltet wird, so die Ergebnisse in RUBYDemenz [23]. Basis dafür ist die Klärung der Ausgangslage und der Entwicklungsziele der Älteren. Als Orientierung eignen sich lernförderliche Bedingungen: Ein Erleben von Selbstbestimmtheit, Eingebundensein, Sinnhaftigkeit erhöht die Lernmotivation, ebendiese werden mit einer Lernbegleitung gemeinsam ermittelt. Ausgehend von Überlegungen zur Alltagsgestaltung werden gewünschte Funktionen des STS zur Ausgangslage für die Erstellung von Lernpfaden, die Gestaltung des Lernens benannt (Motivations-Orientiertes Lernen, MOL [5]).

Dies alles erfordert methodische Kompetenzen bei den Lernbegleitenden, die gelernt sein wollen [23]. Vier Lernbausteine haben sich als zentral herausgestellt [28]:

- Wissen und Erfahrung zum STS, zum *Einsatz* und zur *Handhabung* der Technik,
- Wissen und Erfahrung zu *unterstützenden Gesprächen* mit Nutzenden, Familien, Kooperationspartnern, Forschenden,
- Methodenwissen, -erfahrung und Bereitschaft zur *Mitarbeit in Technikentwicklungsprozessen als Co-Forschende* z. B. zu den Fragen „Wie kann das STS gut eingesetzt werden?“ „Wie wirkt der Einsatz?“ „Was sind geeignete Formen der Beobachtung bei der Nutzung (für die Wechselseitigkeit, Beziehung und Rückmeldung an Entwickelnde)?“
- Wissen zu *ethischen Fragen* und einer reflektierten Auseinandersetzung [23]

Im Projekt wurden die freiwilligen Lernbegleitenden im Rahmen eines Kurses (je Vorbildung 30–40 Unterrichtseinheiten) auf die Aufgabe vorbereitet. In die Fortbildungsprozesse brachten sie ihr Wissen zu Demenz und Pflege in der Familie, ihre darauf bezogenen Kompetenzen, auch bezogen auf die Umsetzung der Technikentwicklung, ein. Zudem waren sie im Rahmen der partizipativen Forschungsprozesse an Erhebungen und Auswertungen beteiligt.

## Diskussion

Die Anforderungen an die Technikbildung im Kontext von STS für die Gestaltung individueller Lernprozesse sowie für das Kursgeschehen erfordern eine Erweiterung des Blicks auf das lernende Subjekt und die Wechselwirkungen im Lernprozess. Ein relationales Verständnis, das die Beziehungsebene einbezieht, steht im Fokus. Es ist wichtig zu beachten, dass bereits in der Aus‑, Fort- und Weiterbildung von Geragog*innen auf diese anspruchsvollen Prozesse eingegangen werden muss. Die Schulung von freiwilligen Lernbegleiter*innen, die Technikaneignungsprozesse moderieren können und die Perspektive der Wechselwirkungen einnehmen, erfordert entsprechendes Know-how bei Geragog*innen. Damit verbunden sind Überlegungen zu Curriculumgestaltung und Methodenvermittlung.

Soziotechnische Systeme für Menschen im höchsten Lebensalter benötigen für eine gelingende Aneignung und Nutzung eine kompetente Lernbegleitung. Diese basiert auch auf der im Forschungsprojekt RUBYDemenz gewonnenen Erkenntnis, dass Lernen in vertrauensvoll erlebten persönlichen Beziehungen besser gelingt: Sie geben Sicherheit und emotionalen Rückhalt, wenn Schwierigkeiten bei der Anwendung TS auftreten. Zugleich haben sie eine Brückenfunktion für soziale Unterstützungssysteme.

Nicht zu unterschätzen ist die Beziehung, die Ältere auch zu TS aufbauen: Technische Systeme werden mit zunehmender Vertrautheit als „persönlich“ wahrgenommen. Dies kann zu positiven Erlebnissen („Wir hatten viel Freude miteinander.“), aber auch zu Enttäuschung führen („Der Roboter spricht nicht mit mir.“). Daraus folgt, dass sich auch Entwickler*innen mit sozialen Folgen von Begegnung mit Robotern befassen müssen.

Ausgehend von den Erfahrungen im Projektkontext und vor dem Hintergrund der partizipativen Forschungsmethodik [23] zeigt sich einmal mehr, dass der Forschungs- und Entwicklungsprozess selbst zu einem Lernprozess werden muss, um TS und STS so zu gestalten, dass sie den bestmöglichen Nutzen erbringen können und auch im Hinblick darauf, dass ihre Aneignung und die damit zusammenhängenden Lernprozesse gelingen [23].

## Fazit für die Praxis

- Als geeignetes interdisziplinäres Lernfeld bieten sich partizipativ angelegte Projekte an, die auf eine enge, geragogisch begleitete Kommunikation von Entwickler*innen und Nutzenden setzen. Dies setzt allerdings voraus, dass Geragogik und geragogische Begleitung von Lernprozessen an Hochschulen als Lehrfach oder Zertifikatskurs angeboten werden und gelernt werden können. Das Angebot der Lernbegleitung für Ältere erfordert eine adäquate Vorbereitung, in der entsprechende Kompetenzen erworben und eingeübt werden können, und das braucht geragogisches Fachpersonal.
- Durch die zunehmende Professionalisierung der Geragogik werden sich neue interdisziplinäre, partizipativ angelegte Praktiken etablieren, die nicht nur die Praxis, sondern auch innovative soziotechnische Forschung und Entwicklung befördern.
